# Metabolic syndrome is associated with an increased incidence of subclinical hypothyroidism – A Cohort Study

**DOI:** 10.1038/s41598-017-07004-2

**Published:** 2017-07-28

**Authors:** Chia-Hsuin Chang, Yi-Chun Yeh, James L. Caffrey, Shyang-Rong Shih, Lee-Ming Chuang, Yu-Kang Tu

**Affiliations:** 1 Institute of Epidemiology and Preventive Medicine, College of Public Health, National Taiwan University, Taipei, Taiwan; 2 Department of Internal Medicine, National Taiwan University Hospital, Taipei, Taiwan; 3 Department of Medicine, College of Medicine, National Taiwan University, Taipei, Taiwan; 4 Institute for Cardiovascular and Metabolic Disease, University of North Texas Health Science Center, Fort Worth, Texas USA

## Abstract

Prior cross-sectional analyses have demonstrated an association between subclinical hypothyroidism and metabolic syndrome and selected components. However, the temporal relation between metabolic syndrome and declining thyroid function remains unclear. In a prospective study, an unselected cohort of 66,822 participants with and without metabolic syndrome were followed. A proportional hazards regression model was used to estimate hazard ratios (HRs) and 95% CIs for hypothyroidism. Exploratory analyses for the relation between components of metabolic syndrome and declining thyroid function were also undertaken. During an average follow-up of 4.2 years, the incident rates for subclinical hypothyroidism were substantially higher in participants who began the study with metabolic syndrome compared with metabolically normal controls. After controlling for risk factors, patients with metabolic syndrome were at a 21% excess risk of developing subclinical hypothyroidism (adjusted HR 1.21; 95% CI 1.03–1.42). When individual components were analyzed, an increased risk of subclinical hypothyroidism was associated with high blood pressure (1.24; 1.04–1.48) and high serum triglycerides (1.18; 1.00–1.39), with a trend of increasing risk as participants had additional more components. Individuals with metabolic syndrome are at a greater risk for developing subclinical hypothyroidism, while its mechanisms and temporal consequences of this observation remain to be determined.

## Introduction

Subclinical hypothyroidism, defined as elevated TSH with free T_4_ concentrations at the lower end of the euthyroid range, affects approximately 4–10% of the general population^1, 2^. Subclinical hypothyroidism has been shown to be associated with more severe coronary and carotid artery disease^3–6^. Furthermore, several large longitudinal studies suggest that mortality and morbidity are higher for patients with both ischemic heart disease and subclinical hypothyroidism, particularly for younger subjects^7–11^. These observations therefore suggest that subclinical hypothyroidism may be a risk factor or a predictive biomarker for cardiovascular diseases.

Although autoimmune disease is often the accepted cause of thyroid dysfunction, it is less clearer which risk factors may predispose or modify hypothyroidism. In this regard it is important to note that many^12–15^, but not all^16, 17^, cross-sectional studies observed that metabolic syndrome and its components, including high blood pressure, elevated triglycerides level, obesity, and insulin resistance, and perhaps high serum cholesterol level, are closely related to subclinical hypothyroidism. Despite these known associations, the temporal relationships between subclinical hypothyroidism and assorted cardiovascular risk factors remain largely unexplored. The aim of this study is to prospectively compare the incidence of subclinical hypothyroidism, among general population with and without metabolic syndrome. The analyses examined further the effect of individual components of the metabolic syndrome on the occurrence of subclinical hypothyroidism.

## Results

**Figure 1:**
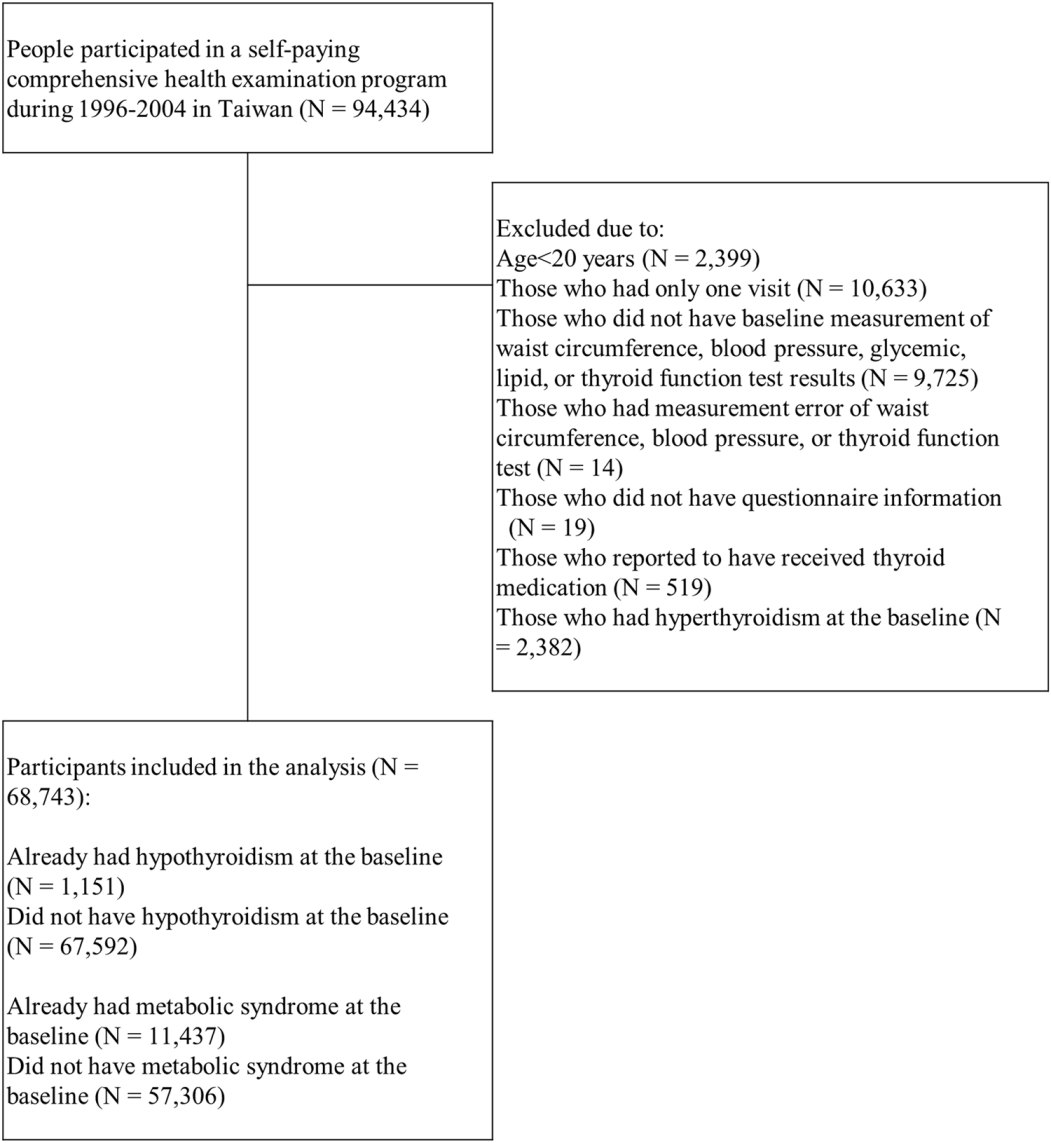
Flow chart for study participants’ enrollment.

The MJ Health Screening database includes a total of 94,434 participants received medical screening in Taiwan between 1996 and 2004. Among them, 9,970 participant with only one medical check-up as well as 68,743 participant with more than one visit met the inclusion criteria. After excluding individuals with only one visit, 68,743 participants were included in our analysis (Fig. 1). Supplementary Table 1 shows the baseline demographic and clinical characteristics of participants with only one visit and those with more than one visit. Compared with individuals with one medical check-up, individual with more than once were younger and had a lower percentage of diabetes, hypertension, hypertriglyceridemia, cholesterol, chronic kidney disease, proteinuria, cardiovascular disease, gout, and arthritis (Supplementary Table 1).

Among them, 52.5% were women, and the mean age was 41.07 years. At the baseline, a total of 11,437 (16.6%) participants had met the criteria for metabolic syndrome and 1,151 (2%) had hypothyroidism, including 92 overt and 1,059 subclinical hypothyroidism. In the cross-sectional analysis, the following variables were significantly related to metabolic syndrome: age ≥ 40 years, male, lower educational level, smoking, drinking, and physical inactivity (Table 1). Meanwhile, hypercholesterolemia, and hyperuricemia were also positively associated with metabolic syndrome, with ORs between 2 to 3. Overt and subclinical hypothyroidism were both significantly associated with metabolic syndrome with OR 1.89 (95% CI: 1.19–2.99) and 1.48 (95% CI: 1.28–1.71) respectively.

**Table 1:** Cross-sectional study: Baseline characteristics of study participants with and without metabolic syndrome (N = 68,743^a,b^).

Variable	Participants no.	Metabolic syndrome				
		No (N = 57,306)		Yes (N = 11,437)		OR (95% CI)
		n	(%)	n	(%)	
Age ≥ 40^a^	68,743	23,149	40.4	8,721	76.3	4.74 (4.52–4.96)
Male^a^	68,743	26,393	46.1	6,286	55.0	1.43 (1.37–1.49)
Lower educational level^a^	68,743	12,661	22.1	5,397	47.2	3.15 (3.02–3.28)
Hypothyroidism^a^	68,743	886	1.6	265	2.3	1.51 (1.32–1.74)
Overt hypothyroidism		67	0.1	25	0.2	1.89 (1.19–2.99)
Subclinical hypothyroidism		819	1.4	240	2.1	1.48 (1.28–1.71)
Hypercholesterolemia (total cholesterol ≥ 240 mg/dL)^a^	68,743	5,342	9.3	2,339	20.5	2.50 (2.37–2.64)
Hyperuricemia (serum uric acid level ≥ 7.2 mg/dl in men or ≥6.0 mg/dl in women)^a^	68,743	15,632	27.3	5,837	51.0	2.78 (2.67–2.90)
Physical inactivity^b^	65,766	26,900	48.9	4,684	43.5	0.80 (0.77–0.84)
Cigarette smoking^b^	62,688	13,789	26.2	3,358	33.3	1.40 (1.34–1.47)
Alcohol consumption^b^	63,476	2,022	3.8	708	6.9	1.88 (1.72–2.05)

^a^Items could be evaluted in all the 68,743 subjects. ^b^Due to missing data in some variables, the participant numbers that could be evaluated were 65,766 for physical inactivity, 62,688 for cigarette smoking, and 63,476 for alcohol consumption.

After excluding participants with prevalent hypothyroidism at the baseline, those with missing information or errors in the records of thyroid function test and those who had ever received thyroid medicine during the follow-up period, there were 66,822 subjects included in the longitudinal analysis. During an average follow-up of 4.2 years, a total of 1,247 new cases of hypothyroidism were identified, including 51 overt and 1,196 subclinical cases. The crude incident rates of subclinical hypothyroidism, but not overt hypothyroidism, were substantially higher in participants with metabolic syndrome than those without metabolic syndrome at the baseline. The crude HR (95% CI) was 1.39 (1.21–1.60), 1.41 (1.23–1.62), and 0.98 (0.46–2.08) for total, subclinical, and overt hypothyroidism respectively (Table 2). After the adjustment of potential risk factors, participants with metabolic syndrome still had a 21% increase in the risk of subclinical hypothyroidism (adjusted HR 1.21; 95% CI 1.03–1.42).

**Table 2:** Follow-up duration, number of incident cases, crude incidence rate, and hazard ratios of hypothyroidism between participants with and without metabolic syndrome at the baseline (N = 66,822^a^).

	Metabolic syndrome	
	No	Yes
N =	55,754	11,068
Follow-up duration (years)		
Total (person-years)	233,363.0	44,057.6
Mean (SD)	4.19 (2.68)	3.98 (2.71)
Median (IQR)	3.47 (1.97–6.15)	3.15 (1.76–5.79)
*Total hypothyroidism*		
Number of incidence cases	989	258
**Crude incidence rate** ^b^	**4.2**	**5.9**
Crude HR (95% CI)	Ref.	1.39 (1.21–1.60)
Adjusted HR (95% CI)^c^	Ref.	1.17 (1.00–1.38)
*Overt hypothyroidism*		
Number of incident cases	43	8
**Crude incidence rate** ^**b**^	**0.2**	**0.2**
Crude HR (95% CI)	Ref.	0.98 (0.46–2.08)
Adjusted HR (95% CI)^c^	Ref.	0.47 (0.17–1.36)
*Subclinical hypothyroidism*		
Number of incident cases	946	250
**Crude incidence rate** ^**b**^	**4.1**	**5.7**
Crude HR (95% CI)	Ref.	1.41 (1.23–1.62)
Adjusted HR (95% CI)^c^	Ref.	1.21 (1.03–1.42)

^a^Excluding participants with prevalent hypothyroidism at the baseline (N = 1,151), those with missing information or measurement error of thyroid function test (N = 631), and those who had ever received undetermined thyroid medicine during the follow-up period (N = 139). ^b^Crude incidence rate: per 1,000 person-years. ^c^Multivariable Cox proportional hazards analyses were adjusted for sex, age group, low educational level, physical inactivity, cigarette smoking, and alcohol consumption.

Regarding individual component of metabolic syndrome, participants with high blood pressure, high serum triglycerides, high waist circumference, and high fasting glucose had a higher crude incidence of subclinical hypothyroidism, but not overt hypothyroidism, than those without any individual component (Table 3). In the analyses of the independent effect associated with each metabolic component, the risk of subclinical hypothyroidism significantly increased with high blood pressure (adjusted HR 1.24; 95% CI 1.04–1.48) and high serum triglycerides level (adjusted HR 1.18; 95% CI 1.00–1.39), but not the other 3 components (Table 4). The additive effect associated with different combinations of the 5 metabolic syndrome components indicated that those who had both high blood pressure and serum triglycerides had a 49% excess risk (adjusted HR 1.49; 95% CI 1.16–1.90). A trend of increasing risk was also noted when participants had additional one, two or three components apart from hypertension and elevated serum triglycerides (Table 4). However, no significantly higher risk was noted for participants with only large waist circumference, high fasting glucose, and low HDL-cholesterol level (adjusted HR 1.06; 95% CI 0.81–1.39).

**Table 3:** Follow-up duration, number of incident cases, and crude incidence rate of hypothyroidism for participants with and without individual component of metabolic syndrome at the baseline (N = 66,822^a^).

Variable	High blood pressure or medicine use		High serum triglycerides or medicine use		High waist circumference		High fasting glucose or medicine use		Low HDL-cholesterol	
	No	Yes	No	Yes	No	Yes	No	Yes	No	Yes
N =	57,578	9,244	52,988	13,834	50,807	16,015	47,836	18,986	40,978	25,844
Follow-up duration (years)										
Total person-years	242,192.5	35,228.1	222,273.7	55,146.9	210,766.9	66,653.7	203,353.8	74,066.8	163,209.5	114,211.1
Mean (SD)	4.21 (2.69)	3.81 (2.59)	4.19 (2.69)	3.99 (2.65)	4.15 (2.64)	4.16 (2.80)	4.25 (2.70)	3.90 (2.62)	3.98 (2.57)	4.42 (2.83)
Median (IQR)	3.48 (1.98–6.18)	3.03 (1.71–5.43)	3.46 (1.97–6.17)	3.21 (1.83–5.79)	3.44 (1.97–6.07)	3.31 (1.84–6.20)	3.57 (1.98–6.26)	3.11 (1.83–5.55)	3.26 (1.92–5.73)	3.70 (1.99–6.76)
*Total hypothyroidism*										
Number of incidence cases	1034	213	954	293	861	386	883	364	717	530
**Crude incidence rate** ^**b**^	**4.3**	**6.0**	**4.3**	**5.3**	**4.1**	**5.8**	**4.4**	**4.9**	**4.4**	**4.6**
*Overt hypothyroidism*										
Number of incidence cases	44	7	36	15	36	15	39	12	32	19
**Crude incidence rate** ^**b**^	**0.2**	**0.2**	**0.2**	**0.3**	**0.2**	**0.2**	**0.2**	**0.2**	**0.2**	**0.2**
*Subclinical hypothyroidism*										
Number of incidence cases	990	206	918	278	825	371	844	352	685	511
**Crude incidence rate** ^**b**^	**4.1**	**5.8**	**4.1**	**5.0**	**3.9**	**5.6**	**4.2**	**4.8**	**4.2**	**4.5**

^a^Excluding participants with prevalent hypothyroidism at the baseline (N = 1,151), and those with missing information or measurement error of thyroid function test (N = 631), and those who had ever received undetermined thyroid medicine during the follow-up period (N = 139). ^b^Crude incidence rate: per 1,000 person-years.

**Table 4:** Hazard ratios of subclinical hypothyroidism comparing participants with different combinations of metabolic syndrome components (N = 66,822^a^).

	Participants no.	Adjusted HR^b^ (95% CI)
**Independent effect**	57,578	
High blood pressure or medicine use		1.24 (1.04–1.48)
High triglycerides or medicine use		1.18 (1.00–1.39)
High waist circumference		1.07 (0.93–1.25)
High fasting glucose or medicine use		1.04 (0.90–1.20)
Low HDL-cholesterol level		0.97 (0.85–1.10)
**Additive effect**		
*Two components*		
**Model 1**: high blood pressure **and** high serum triglycerides	3,675	1.49 (1.16–1.90)
*Three components*		
**Model 2**: high blood pressure **and** high serum triglycerides **and** high waist circumference	2,168	1.50 (1.09–2.05)
**Model 3**: high blood pressure **and** high serum triglycerides **and** high fasting glucose	2,187	1.70 (1.26–2.29)
**Model 4**: high blood pressure **and** high waist circumference **and** high fasting glucose	2,548	1.57 (1.18–2.10)
**Model 5**: high serum triglycerides **and** high waist circumference **and** high fasting glucose	3,322	1.49 (1.14–1.94)
**Model 6**: high waist circumference **and** high fasting glucose **and** low HDL-cholesterol level	3,873	1.06 (0.81–1.39)
*Four components*		
**Model 7**: high blood pressure **and** high serum triglycerides **and** high waist circumference **and** high fasting glucose	1,382	1.86 (1.30–2.65)
*Five components*		
**Model 8**: high blood pressure **and** high serum triglycerides **and** high waist circumference **and** high fasting glucose **and** low HDL-cholesterol level	863	1.55 (0.98–2.45)

^a^Excluding participants with prevalent hypothyroidism at the baseline (N = 1,151), and those with missing information or measurement error of thyroid function test (N = 631), and those who had ever received undetermined thyroid medicine during the follow-up period (N = 139). ^b^Multivariable Cox proportional hazards analyses were adjusted for sex, age group, low educational level, physical inactivity, cigarette smoking, and alcohol consumption. Reference group: normal value for all 5 components.

For sensitivity analyses on the associations between metabolic syndrome and subclinical hypothyroidism, we excluded physical inactivity as a covariate for adjustment, additionally controlled for hypercholesterolemia at the baseline, undertook competing risk analysis by considering overt hypothyroidism and hyperthyroidism as competing risks, and excluded participants who were possibly to have treated hypothyroidism during the follow-up period, found no substantial differences (Supplement Table 2). In the stratified analyses aimed to evaluate whether risks were modified by baseline characteristics, the risks of new hypothyroidism associated with metabolic syndrome were similar for men and women, and for those aged < 40 and ≥ 40 years in the beginning of the study (Supplement Table 3).

## Discussion

In this prospective cohort study, participants with metabolic syndrome were associated with a significantly increased risk of developing subclinical hypothyroidism during an average follow-up of 4 years compared to those without metabolic syndrome at the baseline. High blood pressure and high serum triglycerides may play a more important role than other metabolic syndrome components in developing subclinical hypothyroidism, although a trend of increasing risks was observed, if participants had additional components.

While prior cross-sectional studies have reported both functional and morphological changes in thyroid gland among metabolic syndrome patients^18^, this longitudinal study clearly demonstrates the temporal relation between these two conditions. Metabolic syndrome appears to be a risk factor for subclinical hypothyroidism. In the context of higher cardiovascular risk associated with subclinical hypothyroidism and the metabolic syndrome, the current analyses suggests that thyroid dysfunction may be one intermediate factor between metabolic syndrome and cardiovascular disease. These cross-sectional associations are clearly multifactorial including increased inflammation, pro-thrombosis, impaired fibrinolysis, endothelial dysfunction^19^, insulin sensitivity and endothelium-dependent vasodilation^20, 21^. In a retrospective study analyzing the United Kingdom General Practitioner Research Database, Razvi and colleagues found treatment of subclinical hypothyroidism with levothyroxine was associated with fewer ischemic heart disease events in the younger individuals, supporting a beneficial effect of thyroid hormone on cardiovascular risk factors in hypothyroid patients^22^. Whether thyroid dysfunction has a major direct effect, or is just an intermediate mediator, or simply a “bystander” in these many pathways leading to coronary atherosclerosis needs to be continuously monitored. Thus, exploring the reverse relationship between metabolic syndrome and thyroid dysfunction as well as the temporal sequence of association of metabolic syndrome, thyroid dysfunction, and cardiovascular diseases may be a worthwhile topic for future research. Furthermore, the efficacy of hypothyroidism medicine to prevent the development of metabolic syndrome should also be investigated in future studies.

Previous studies using factor analysis or structural equation modeling to understand the underlying structural of the co-occurrence of metabolic risk factors suggest that metabolic syndrome is represented primarily by “insulin resistance” and “obesity”, followed by “lipids”, and the “blood pressure”. Further, “insulin resistance/hyperinsulinemia” may be the common unifying factor that links all the core components^23–26^. Despite blood pressure being loosely associated with the central features of metabolic syndrome, the current analyses suggest that blood pressure was the metabolic factor that most strongly associated with the occurrence of thyroid hypofunction. This relationship may reflect the observation that hypertension is the most common (and perhaps earliest) manifestation within the myriad of at-risk phenotypes associated with atherosclerotic cardiovascular disease. Furthermore, among all the possible pathological pathways leading to hypertension^27^, several share a link with hypothyroidism including changes in circulating catecholamines, disturbances in the renin-angiotensin-aldosterone system, and increased peripheral vascular resistance^28, 29^. Although it is widely accepted that there is a close relation between hypercholesterolemia and overt hypothyroidism, our analyses suggest high serum triglycerides be a significant perhaps independent factor for increasing the risk of subclinical hypothyroidism. This effect remains unchanged even after controlling for high serum cholesterol. Evidence suggests that obesity results in fat accumulation in the thyroid gland in humans and a mouse model. This may affect the thyroid hormone production and result in hypothyroidism, as suggested by studies in obese mice^30^. Our study finding suggests that the association between thyroid hypofunction and high serum triglyceride level was stronger than that of high waist circumference. The connection between the metabolic syndrome components and the development of thyroid dysfunction merit further research to elaborate the underlying mechanisms.

It is well known that hypertension is often induced by excess salt intake and it is possible that people with hypertension may have excess ingestion of iodized salt. Increasing evidence suggests that a higher population iodine intake is associated with an increased level of serum TSH and more cases of hypothyroidism in the population^31, 32^. Excess iodine ingestion which results in thyroid dysfunction in susceptible individuals, however, is nevertheless well-tolerated in most people^33^. In Taiwan, the mandatory salt iodization program was implemented since 1967 but changed to voluntary salt iodization since 2003^34^. We could not further examine whether the observed association between hypertension and hypothyroidism is caused by an excess intake of iodized salt owing to that the food frequency questionnaire does not specifically assess salt and iodine intake. Meanwhile, it is well known that thyroid hormones play an important role in lipid metabolism and there were many cross-sectional studies reporting the association between hypertriglyceridemia and hypothyroidism^35^. However, limited studies investigate the reverse, that is, the effect of high serum triglycerides level on thyroid gland morphology and function. In an animal study, Han and colleagues demonstrated that excess iodine combined with high-fat diet could cause damage to thyroid glands and lead to thyroid hormone disorder in mice^36^. In another study, Shao and colleagues found that rats fed a high-fat lard diet for 24 weeks had significantly increased serum triglyceride levels in both the serum and thyroid tissue, decreased serum total T4 and free T4 levels in parallel with elevated serum TSH levels, and altered macro and micro morphology of the thyroid gland^37^. In the present study, we could not further examine whether the observed association between hypertriglyceridemia and hypothyroidism is due to high fat intake. Further researches are warranted to evaluate the potential role of dietary factors that may influence the occurrence of thyroid hypofunction in the susceptible population.

The main strength of this study is that a large number of participants had been observed prospectively for several years. Participants received thyroid function tests and metabolic risk factor measurements at regular intervals such that detection bias due to differential lengths of follow-up could be minimized. Educational level and important lifestyle factors were controlled in the analysis to partition confounding effects due to these factors. Similar results following several sensitivity analyses adjusting for different risk factors or after taking competing risk into consideration suggest the current study findings are quite robust. There are also some limitations in this study. First, in this study, the follow-up duration may not be sufficiently long enough to determine the full clinical significance of subclinical thyroid dysfunction related to metabolic syndrome. Whether these cases associated with metabolic syndrome will progress to overt hypothyroidism, remain in the same status, or return to a euthyroid state needs to be examined in a longer prospective study. Second, the definition of metabolic syndrome in the present study was based on The Third Report of the National Cholesterol Education Program Expert Panel (NCEP) on Detection, Evaluation, and Treatment of High Blood Cholesterol in Adults (Adult Treatment Panel III) with ethnic-specific values for waist circumference in order to be easy to compare findings with other research findings. The analyses did not examine the degree of deviation from normal or the most optimal cut-off point for individual metabolic component for the development of subsequent subclinical hypothyroidism. Third, the definition of overt and subclinical hypothyroidism was based on a single TSH and T4 value, without taking an age-adjusted TSH range into account or doubly confirmed by TSH elevation. It could probably introduce misclassification; however, this is a non-differential misclassification which biased the results toward the null. Finally, since the study population came from fee-paying participants, whether the results can be generalized to a less selected and/or less financially capable population remains to be determined.

In conclusion, our analyses suggest metabolic syndrome increases the risk of acquiring subclinical hypothyroidism, a condition routinely associated with a significantly increased risk of atherosclerotic heart disease morbidity and mortality. The mechanisms and clinical consequences of the observed relations among metabolic risk factors and thyroid hypofunction needed to be explored in the future studies.

## Methods

### Data source and study population

Potential participants for this prospective study came from a total of 94,434 individuals who participated in a self-paying comprehensive health examination program offered by a private firm (MJ Health Management Institute, Taiwan) between 1996 and 2004^38^. The protocol was approved by the Research Ethics Committee in Leeds Institute of Genetics, Health and Therapeutics at the University of Leeds, Leeds, UK. All methods were performed in accordance with the relevant guidelines and regulations. The data used in this study were held and approved by MJ Health Management Institute, Taiwan. To comply with regulations related to the privacy of personal electronic data, the identity of each patient was encrypted and all data was analyzed anonymously. Hence, the IRB granted a waiver of informed consent.

Participants were excluded if 1) were not adequately followed during the study period; 2) initial or follow up test data were missing or in error; 3) questionnaires were missing; 4) reported to have received thyroid medicine at the baseline; and 5) began the study with diagnosed thyroid dysfunction. In the analysis of the association between metabolic syndrome and incident hypothyroidism, those who reported to have ever received thyroid medication during the follow-up period were excluded because thyroid hyper- or hypo-function could not be determined by questionnaire.

### Data collection

In addition to a self-administered questionnaire for education level, lifestyle factors, and past medical history, each participant received a standard physical examination including anthropomorphic measurements, and blood and urine analyses. Overnight fasting blood and first morning voided urine were collected and analyzed.

### Definition of metabolic syndrome and hypothyroidism

Metabolic syndrome was defined by at least 3 of the following 5 components: blood pressure ≥130/85 mm Hg (or receiving drug therapy for hypertension), serum triglycerides ≥150 mg/dL (or receiving drug therapy for hypertriglyceridemia),fasting glucose ≥100 mg/dL (or receiving drug therapy for hyperglycemia), HDL-cholesterol <40 mg/dL in men or <50 mg/dL in women, and waist circumference ≥90 cm (35 inch) in men or ≥80 cm (32 inch) in women.

Hypothyroidism was defined based on symptoms and laboratory values. Overt hypothyroidism was assumed if TSH was greater than 5 μU/ml and T4 less than 4.5 μg/dl. Subclinical hypothyroidism was defined as elevated TSH values (>5 μU/ml) but having T4 values between 4.5 and 12 μg/dl^39^.

### Statistical analyses

Baseline characteristics for all study participants were summarized. In the cross-sectional analysis, a logistic regression model was used to estimate crude odds ratios (ORs) and 95% confidence intervals (CIs) for the risk factors potentially related to metabolic syndrome. In the subsequent cohort analysis, participants with and without metabolic syndrome were followed from the first visit to the first report of hypothyroidism, or the last visit with available blood test results. The crude incident rates for total hypothyroidism, overt, and subclinical hypothyroidism were calculated. Cox proportional hazards regression models were used to estimate hazard ratios (HRs) and 95% CIs for incident hypothyroidism with the adjustment of important risk factors, such as age, sex, cigarette smoking, alcohol consumption, physical inactivity, and low educational level. The proportional hazard assumption was examined by plotting the log minus log survival curves and survival times against cumulative survival. To further explore the independent and additive effect associated with each component, further analyses were conducted to evaluate whether the risk of hypothyroidism increased with the greater numbers of metabolic syndrome components, and which combinations of metabolic syndrome components were associated with the highest risk for hypothyroidism. Several sensitivity analyses including: 1) not controlling for physical inactivity, 2) additionally controlling for hypercholesterolemia at the baseline, 3) by competing risk analysis, and 4) excluding participants who were possibly to have treated hypothyroidism at the last follow-up visit, were performed to evaluate their influence on the results.

Stratified analyses were performed to evaluate whether baseline characteristics modified the risks. Participants were stratified according to sex (men, women) and age (<40, ≥40 years). A likelihood ratio test was conducted to evaluate the interaction between gender, age, and metabolic syndrome component for possible effect modification. Two-sided *p* value < 0.05 was considered to be statistically significant. All statistical analyses were performed with SAS 9.4 (SAS Institute, Cary, NC).

## Electronic supplementary material

Supplementary dataset
