# Supplementary material for: Metabolic syndrome is associated with an increased incidence of subclinical hypothyroidism – A Cohort Study
Source: Sci Rep. 2017 Jul 28;7:6754. doi: 10.1038/s41598-017-07004-2 (PMC5533753; doi:10.1038/s41598-017-07004-2)
Supplement: Supplementary file 1 — Supplementary dataset [file 41598_2017_7004_MOESM1_ESM.doc]

**Supplementary data to:**

**Metabolic syndrome is associated with an increased incidence of subclinical hypothyroidism – A Cohort Study**

Chia-Hsuin Chang, Yi-Chun Yeh, James L. Caffrey, Shyang-Rong Shih, Lee-Ming Chuang, Yu-Kang Tu

**Supplementary Table 1. Baseline characteristics of study participants with only one visit and more than one visit**

|  | *Only one visit ^a^* | |  | *More than one visit* | |  |
| --- | --- | --- | --- | --- | --- | --- |
| *Variable* | *n* | *(%)* |  | *n* | *(%)* | *P value* |
| **Total** | **9970** | **(100.0)** |  | **68743** | **(100.0)** |  |
| **Age** |  |  |  |  |  |  |
| Mean(SD) | 44.22 (14.57) | |  | 41.07 (13.15) | | <0.0001 |
| Median (Q1,Q3) | 41.00 (33.00,56.00) | |  | 38.00 (31.00,50.00) | |  |
| **Age≥40** |  |  |  |  |  |  |
| No | 4514 | (45.3) |  | 36873 | (53.6) | <0.001 |
| Yes | 5456 | (54.7) |  | 31870 | (46.4) |  |
| **Gender** |  |  |  |  |  |  |
| Female | 5243 | (52.6) |  | 36064 | (52.5) |  |
| Male | 4727 | (47.4) |  | 32679 | (47.5) | 0.81 |
| **Lower educational level** |  |  |  |  |  |  |
| No | 6210 | (62.3) |  | 50685 | (73.7) | <0.001 |
| Yes | 3760 | (37.7) |  | 18058 | (26.3) |  |
| **Thyroid dysfunction** |  |  |  |  |  |  |
| Euthyroidism | 9804 | (98.3) |  | 67592 | (98.3) | 0.95 |
| Hypothyroidism | 166 | (1.7) |  | 1151 | (1.7) |  |
| **High fasting glucose or medicine use** |  |  |  |  |  |  |
| No | 6857 | (68.8) |  | 49211 | (71.6) | <0.001 |
| Yes | 3113 | (31.2) |  | 19532 | (28.4) |  |
| **High blood pressure or medicine use** |  |  |  |  |  |  |
| No | 8345 | (83.7) |  | 59172 | (86.1) | <0.001 |
| Yes | 1625 | (16.3) |  | 9571 | (13.9) |  |
| **Hypertriglyceridemia or medicine use** |  |  |  |  |  |  |
| No | 7657 | (76.8) |  | 54430 | (79.2) | <0.001 |
| Yes | 2313 | (23.2) |  | 14313 | (20.8) |  |
| **Low HDL Cholesterol** |  |  |  |  |  |  |
| No | 5403 | (54.2) |  | 42171 | (61.3) | <0.001 |
| Yes | 4567 | (45.8) |  | 26572 | (38.7) |  |
| **High waist circumference** |  |  |  |  |  |  |
| No | 6797 | (68.2) |  | 52227 | (76.0) | <0.001 |
| Yes | 3173 | (31.8) |  | 16516 | (24.0) |  |
| **High serum cholesterol** |  |  |  |  |  |  |
| No | 8748 | (87.7) |  | 61062 | (88.8) | 0.0014 |
| Yes | 1222 | (12.3) |  | 7681 | (11.2) |  |
| **Chronic kidney disease stage 2-5** |  |  |  |  |  |  |
| No | 4053 | (40.7) |  | 31645 | (46.0) | <0.001 |
| Yes | 5917 | (59.3) |  | 37097 | (54.0) |  |
| Missing |  |  |  | 1 |  |  |
| **Total proteinuria** |  |  |  |  |  |  |
| No | 8675 | (90.8) |  | 61330 | (93.2) | <0.001 |
| Yes | 880 | (9.2) |  | 4471 | (6.8) |  |
| Missing | 415 |  |  | 2942 |  |  |
| **Physical inactivity** |  |  |  |  |  |  |
| No | 4934 | (53.2) |  | 34176 | (52.0) | 0.02 |
| Yes | 4336 | (46.8) |  | 31583 | (48.0) |  |
| Missing | 700 |  |  | 2984 |  |  |
| **Smoking** |  |  |  |  |  |  |
| No | 5811 | (67.1) |  | 45531 | (72.7) | <0.001 |
| Yes | 2853 | (32.9) |  | 17138 | (27.3) |  |
| Missing | 1306 |  |  | 6074 |  |  |
| **Drinking** |  |  |  |  |  |  |
| No | 8276 | (94.8) |  | 60735 | (95.7) | <0.001 |
| Yes | 456 | (5.2) |  | 2730 | (4.3) |  |
| Missing | 1238 |  |  | 5278 |  |  |
| **Obesity** |  |  |  |  |  |  |
| No | 9519 | (95.5) |  | 66560 | (96.8) | <0.001 |
| Yes | 449 | (4.5) |  | 2180 | (3.2) |  |
| Missing | 2 |  |  | 3 |  |  |
| **Sleep time >6 hours/night** |  |  |  |  |  |  |
| No | 1961 | (20.7) |  | 12682 | (19.0) | <0.001 |
| Yes | 7523 | (79.3) |  | 53895 | (81.0) |  |
| Missing | 486 |  |  | 2166 |  |  |
| **Sugar drinks >1 cup/week** |  |  |  |  |  |  |
| No | 5679 | (66.8) |  | 34428 | (55.2) | <0.001 |
| Yes | 2819 | (33.2) |  | 27913 | (44.8) |  |
| Missing | 1472 |  |  | 6402 |  |  |
| **Cardiovascular disease** |  |  |  |  |  |  |
| No | 9640 | (96.7) |  | 66918 | (97.3) | <0.001 |
| Yes | 330 | (3.3) |  | 1825 | (2.7) |  |
| **Asthma** |  |  |  |  |  |  |
| No | 9692 | (97.2) |  | 66775 | (97.1) | 0.68 |
| Yes | 278 | (2.8) |  | 1968 | (2.9) |  |
| **Peptic ulcer disease** |  |  |  |  |  |  |
| No | 9187 | (92.1) |  | 63046 | (91.7) | 0.14 |
| Yes | 783 | (7.9) |  | 5697 | (8.3) |  |
| **Hepatitis or Liver cirrhosis** |  |  |  |  |  |  |
| No | 9144 | (91.7) |  | 62935 | (91.6) | 0.58 |
| Yes | 825 | (8.3) |  | 5801 | (8.4) |  |
| Missing | 1 |  |  | 7 |  |  |
| **Gout** |  |  |  |  |  |  |
| No | 9544 | (95.7) |  | 66069 | (96.1) | 0.07 |
| Yes | 426 | (4.3) |  | 2674 | (3.9) |  |
| **Arthritis** |  |  |  |  |  |  |
| No | 9352 | (93.8) |  | 65227 | (94.9) | <0.001 |
| Yes | 618 | (6.2) |  | 3516 | (5.1) |  |
| **Family history of hypertension** |  |  |  |  |  |  |
| No | 7374 | (74.0) |  | 47576 | (69.2) | <0.001 |
| Yes | 2596 | (26.0) |  | 21167 | (30.8) |  |
| **Family history of diabetes** |  |  |  |  |  |  |
| No | 8199 | (82.2) |  | 54137 | (78.8) | <0.001 |
| Yes | 1771 | (17.8) |  | 14606 | (21.2) |  |
| **Family history of cerebrovascular disease** |  |  |  |  |  |  |
| No | 9357 | (93.9) |  | 62593 | (91.1) | <0.001 |
| Yes | 613 | (6.1) |  | 6150 | (8.9) |  |
| **Family history of cardiovascular disease** |  |  |  |  |  |  |
| No | 9069 | (91.0) |  | 61152 | (89.0) | <0.001 |
| Yes | 901 | (9.0) |  | 7591 | (11.0) |  |

^a^ Excluding participants with missing information of fasting glucose (N=6), blood lipid (N=94), thyroid function test (N=14), questionnaire (N=6), measurement error of thyroid function (N=4), those who reported to receive thyroid medication (N=85), and participants with hyperthyroidism (N=454).

**Supplementary Table 2**. Adjusted hazard ratios of incident hypothyroidism comparing participants with and without metabolic syndrome by several sensitivity analyses (N = 66,822)

|  | *Total hypothyroidism* |  | *Overt hypothyroidism* |  | *Subclinical hypothyroidism* |
| --- | --- | --- | --- | --- | --- |
|  | *Adjusted HR (95%CI)* |  | *Adjusted HR (95%CI)* |  | *Adjusted HR (95%CI)* |
| **Model 1:** Controlled for different risk factors ^a^ |  |  |  |  |  |
| Metabolic syndrome |  |  |  |  |  |
| No | Ref. |  | Ref. |  | Ref. |
| Yes | 1.16 (0.99-1.36) |  | 0.42 (0.15-1.19) |  | 1.20 (1.02-1.41) |
| **Model 2:** Controlled for different risk factors ^b^ |  |  |  |  |  |
| Metabolic syndrome |  |  |  |  |  |
| No | Ref. |  | Ref. |  | Ref. |
| Yes | 1.17 (1.00-1.37) |  | 0.48 (0.17-1.38) |  | 1.20 (1.02-1.42) |
| **Model 3:** Competing risk analyses ^c^ |  |  |  |  |  |
| Metabolic syndrome |  |  |  |  |  |
| No | Ref. |  | Ref. |  | Ref. |
| Yes | 1.18 (1.00-1.38) |  | 0.47 (0.16-1.41) |  | 1.21 (1.03-1.43) |
| **Model 4**: Excluding participants who were possibly to have treated hypothyroidism during the follow-up period ^c, d^ |  |  |  |  |  |
| Metabolic syndrome |  |  |  |  |  |
| No | Ref. |  | Ref. |  | Ref. |
| Yes | 1.17 (0.99-1.37) |  | 0.14 (0.02-1.01) |  | 1.21 (1.03-1.43) |
| **Model 5:** Excluding participants who reported to receive antidiabetic agent during the follow-up period ^c, e^ |  |  |  |  |  |
| Metabolic syndrome |  |  |  |  |  |
| No | Ref. |  | Ref. |  | Ref. |
| Yes | 1.23 (1.04-1.45) |  | 0.53 (0.18-1.52) |  | 1.27 (1.07-1.50) |
| **Model 6:** Excluding participants who reported to receive antidiabetic, antihypertensive, and antihyperlipidemic agnets during the follow-up period ^c, e, f^ |  |  |  |  |  |
| Metabolic syndrome |  |  |  |  |  |
| No | Ref. |  | Ref. |  | Ref. |
| Yes | 1.18 (0.97-1.43) |  | 0.21 (0.03-1.53) |  | 1.23 (1.01-1.49) |

^a^ Multivariable Cox proportional hazards analyses adjusted for sex, age group, cigarette smoking, alcohol consumption, and low educational level

^b^ Multivariable Cox proportional hazards regression model adjusted for sex, age group, cigarette smoking, alcohol consumption, physical inactivity, low educational level, and hypercholesterolemia

^c^ Multivariable Cox proportional hazards regression model adjusted for sex, age group, cigarette smoking, alcohol consumption, physical inactivity, and low educational level.

^d^ Excluding participants who had low thyroid function test result and reported to receive thyroid medicine at the last follow-up visit (N = 214)

^e^ Excluding participants who reported to receive anti-diabetic agents at the last follow-up visit (N = 1,848)

^f^ Excluding participants who reported to receive anti-hypertensive and antihyperlipidemic agents at the last follow-up visit (N = 4,796)

**Supplementary Table 3.** Hazard ratios of subclinical hypothyroidism comparing participants with and without metabolic syndrome based on sex, and age (N = 66,822)

| *Variable* | *Men (n=31,936)* | | | *Women (n=34,886)* | | | *p value for interaction between gender and subclinical hypothyroidism* |
| --- | --- | --- | --- | --- | --- | --- | --- |
|  | *Event no.* | *Crude HR* | *Adjusted HR* ^a^ | *Event no.* | *Crude HR* | *Adjusted HR* ^a^ |  |
| **Metabolic syndrome** | 357 | 1.76 (1.40-2.21) | 1.42 (1.11-1.83) | 839 | 1.39 (1.16-1.66) | 1.09 (0.88-1.34) | 0.10 |
| High blood pressure or medicine use | 357 | 1.61 (1.26-2.07) | 1.39 (1.06-1.81) | 839 | 1.57 (1.30-1.90) | 1.25 (1.01-1.55) | 0.55 |
| High serum triglycerides or medicine use | 357 | 1.47 (1.19-1.83) | 1.29 (1.02-1.62) | 839 | 1.51 (1.27-1.80) | 1.19 (0.97-1.46) | 0.62 |
| High waist circumference | 357 | 1.44 (1.14-1.81) | 1.28 (1.99-1.64) | 839 | 1.31 (1.14-1.52) | 1.08 (0.91-1.29) | 0.25 |
| High fasting glucose or medicine use | 357 | 1.40 (1.14-1.74) | 1.16 (0.92-1.46) | 839 | 1.25 (1.07-1.46) | 1.05 (0.88-1.26) | 0.51 |
| Low HDL-cholesterol | 357 | 0.90 (0.73-1.12) | 0.88 (0.70-1.11) | 839 | 1.08 (0.94-1.24) | 1.08 (0.93-1.26) | 0.14 |
|  |  |  |  |  |  |  |  |
|  | *Age < 40 (n=36,020)* | | | *Age ≥ 40 (n=30,802)* | | | *p value for interaction between age groups and subclinical hypothyroidism* |
| *Variable* | *Event no.* | *Crude HR* | *Adjusted HR* ^a^ | *Event no.* | *Crude HR* | *Adjusted HR* ^a^ |  |
| **Metabolic syndrome** | 492 | 1.15 (0.83-1.58) | 1.28 (0.89-1.84) | 704 | 1.17 (1.00-1.38) | 1.19 (0.99-1.43) | 0.74 |
| High blood pressure or medicine use | 492 | 1.03 (0.67-1.56) | 1.46 (0.95-2.25) | 704 | 1.21 (1.02-1.43) | 1.28 (1.06-1.53) | 0.57 |
| High serum triglycerides or medicine use | 492 | 1.07 (0.83-1.37) | 1.36 (1.03-1.80) | 704 | 1.10 (0.94-1.30) | 1.18 (0.98-1.41) | 0.39 |
| High waist circumference | 492 | 1.19 (0.94-1.51) | 1.27 (0.99-1.64) | 704 | 1.21 (1.04-1.41) | 1.08 (0.91-1.28) | 0.30 |
| High fasting glucose or medicine use | 492 | 0.96 (0.75-1.22) | 1.08 (0.83-1.41) | 704 | 0.99 (0.85-1.16) | 1.10 (0.93-1.30) | 0.93 |
| Low HDL-cholesterol | 492 | 1.08 (0.91-1.30) | 1.02 (0.84-1.23) | 704 | 1.01 (0.87-1.17) | 1.02 (0.86-1.20) | 0.99 |

^a^ Multivariable Cox proportional hazards regression model adjusted for sex, age group, cigarette smoking, alcohol consumption, physical inactivity, and low educational level

**Supplementary Table 4.** Follow-up duration, number of incident cases, crude incidence rate, and hazard ratios of metabolic syndrome among participants with eruthyroidism and hypthyroidism at the baseline (N = 57,058 ^a^).

|  | *Euthyroidism* | *Total hypothyroidism* | *Subclinical hypothyroidism* | *Overt hypothyroidism* |
| --- | --- | --- | --- | --- |
| N = | 56,176 | 882 | 815 | 67 |
| Follow-up duration (years) |  |  |  |  |
| Total (person-years) | 229,105.0 | 3,486.1 | 3272.2 | 213.9 |
| Mean (SD) | 4.08(2.65) | 3.95(2.63) | 4.01(2.64) | 3.19(2.40) |
| Median (IQR) | 3.33(1.93, 5.98) | 3.15(1.88, 5.77) | 3.20(1.91, 5.95) | 2.26(1.34, 4.27) |
| ***Metabolic syndrome*** |  |  |  |  |
| Number of incidence cases | 6,713 | 122 | 113 | 9 |
| **Crude incidence rate ^b^** | **29.3** | **35.0** | **34.5** | **42.1** |
| Crude HR(95%CI) | Ref. | 1.20 (1.01-1.44) | 1.19 (0.99-1.43) | 1.47 (0.77-2.82) |
| Adjusted HR(95%CI) ^c^ | Ref. | 1.10 (0.91-1.35) | 1.07 (0.87-1.32) | 1.65 (0.82-3.29) |

^a^ Excluding participants with prevalent metabolic syndrome at the baseline (N = 11,437), those with missing information or measurement error of metabolic profiles during follow-up period (N = 248).

^b^ Crude incidence rate: per 1,000 person-years.

^c^ Multivariable Cox proportional hazards regression model adjusted for sex, age group, low educational level, physical inactivity, cigarette smoking, and alcohol consumption.

**Supplementary Table 5**. Adjusted hazard ratios of incident metabolic syndrome comparing participants with hypothyroidism with euthyroidism (N = 57,058)

|  | *Metabolic syndrome* |
| --- | --- |
|  | *Adjusted HR (95%CI)* |
| **Model 1**: Excluding participants who reported to receive anti-diabetic agents at baseline ^a, b^ |  |
| Total hypothyroidism | 1.09 (0.89-1.34) |
| Subclinical hypothyroidism | 1.07 (0.87-1.32) |
| Overt hypothyroidism | 1.49 (0.71-3.12) |
| **Model 2:** Excluding participants who reported to receive antidiabetic, antihypertensive and antihyperlipidemic agents at baseline ^a, b, c^ |  |
| Total hypothyroidism | 1.09 (0.88-1.35) |
| Subclinical hypothyroidism | 1.06 (0.85-1.32) |
| Overt hypothyroidism | 1.63 (0.78-3.42) |

^a^ Multivariable Cox proportional hazards regression model adjusted for sex, age group, cigarette smoking, alcohol consumption, physical inactivity, and low educational level.

^b^ Excluding participants who reported to receive antidiabetic medicine at baseline (N = 404)

^c^ Excluding participants who reported to receive antihypertensive and antihyperlipidemic medicines at baseline (N = 1,258)
